# Chemical Modification of Sweet Potato β-amylase by Mal-mPEG to Improve Its Enzymatic Characteristics

**DOI:** 10.3390/molecules23112754

**Published:** 2018-10-24

**Authors:** Xinhong Liang, Wanli Zhang, Junjian Ran, Junliang Sun, Lingxia Jiao, Longfei Feng, Benguo Liu

**Affiliations:** School of Food Science, Henan Institute of Science and Technology, Xinxiang 453003, China; liangxinhong2005@163.com (X.L.); zwl6996468@126.com (W.Z.); ranjunjian@126.com (J.R.); jiaolingxia@163.com (L.J.); flf18738380903_007@163.com (L.F.); zzgclbg@126.com (B.L.)

**Keywords:** sweet potato β-amylase (SPA) 2, methoxy polyethylene glycol maleimide (Mal-mPEG) 3, chemical modification 4, enzymatic characteristics

## Abstract

The sweet potato β-amylase (SPA) was modified by 6 types of methoxy polyethylene glycol to enhance its specific activity and thermal stability. The aims of the study were to select the optimum modifier, optimize the modification parameters, and further investigate the characterization of the modified SPA. The results showed that methoxy polyethylene glycol maleimide (molecular weight 5000, Mal-mPEG5000) was the optimum modifier of SPA; Under the optimal modification conditions, the specific activity of Mal-mPEG5000-SPA was 24.06% higher than that of the untreated SPA. Mal-mPEG5000-SPA was monomeric with a molecular weight of about 67 kDa by SDS-PAGE. The characteristics of Mal-mPEG5000-SPA were significantly improved. The K_m_ value, V_max_ and *Ea* in Mal-mPEG5000-SPA for sweet potato starch showed that Mal-mPEG5000-SPA had greater affinity for sweet potato starch and higher speed of hydrolysis than SPA. There was no significant difference of the metal ions’ effect on Mal-mPEG5000-SPA and SPA.

## 1. Introduction

β-amylase (E.C. 3.2.1.2) is an exo-type saccharifying enzyme that can act on the α-1,4 glucosidic bonds and cleave off maltose units at the non-reducing end of starch molecules [1]. The cleave-off process is accompanied by Walden inversion that turns the product from α-maltose into β-maltose [2,3]. β-amylase exists widely in higher plants such as sweet potato, barley, wheat, and soybea [4], and is mainly applied in the industrial process including food, fermentation, textiles, pharmaceuticals, etc. [5,6]. Sweet potato β-amylase (SPA) is an important component of protein in sweet potato tubers next only to sporamin, and is primarily obtained by extraction and separation from the waste water of the sweet potato starch production [7]. As a bio-active biomacromolecule, SPA’s biological activity and thermal stability are among the key factors that limit its application in the food industry.

Chemical modification of molecules is an effective means to increase enzymatic stability and biological activity. It can also effectively prolong the half-life of enzymes. Polyethylene glycol (PEG) is a good non-irritant amphipathic organic solvent without immunogenicity, antigenicity and toxicity [8,9]. The studies made by Abuehowski et al. as early as 1977 showed that proteins modified by PEG were of greater efficacy than the unmodified ones [10]. Meanwhile, through enzyme modification, the antigenicity of particular enzymes can be reduced or eliminated, and the enzymatic stability can be enhanced. Therefore, the techniques of PEG-modified proteins have been developing rapidly [11,12]. Presently, over 10 types of PEG-modified proteins have been certified by the US Food and Drug Administration [13]. However, due to deficiencies such as frequent occurrence of crosslinking and agglomeration of -OH at both ends of PEG, its usage in protein modification is limited.

With similar properties to PEG, methoxy polyethylene glycol (mPEG) also works as a protein modifier with the active hydroxyl group at one end of PEG being blocked by the methoxy group. Modification through mPEG can change the relevant biological characteristics of enzymes or proteins, including hydrophobicity, surface charge, stability and water solubility [13,14,15]. It is reported that the activity, thermal stability and pH stability of enzymes modified by mPEG can be enhanced. Using mPEG to modify neutral protease changed the thermal stability of the enzyme, and the modified enzyme showed higher affinity for substrate [16]. Fang et al. [17] modified phospholipase C by using mPEG-succinimidyl succinate ester. The results indicated an increase by three times in the catalytic efficiency of the modified phospholipase C, and also higher thermal stability. Daba et al. [18] adopted glutaraldehyde (GA), mPEG chlorotriazine and trinitro-benzene-sulfonic acid (TNBS) to modify β-amylase in malt. The result indicated that mPEG chlorotriazine enhanced the enzymatic activity and thermal stability of β-amylase in malt. However, there are very few reports about mPEG modified SPA.

Methoxy polyethylene glycol N-hydroxylsuccinimide ester (NHS-mPEG5000, NHS-mPEG20000), Methoxy polyethylene glycol tosylate (Ts-mPEG5000, Ts-mPEG5000, Ts-mPEG10000, Ts-mPEG20000) and Methoxy polyethylene glycol maleimide (Mal-mPEG5000) were adopted in this study for chemical modification on SPA to screen the optimum modifier. The response surface method was applied to optimize the molar ratio of the optimum modifier to SPA, as well as the modification temperature, pH value and other parameters. The enzymatic properties of the modification enzyme under optimal parameters were studied to improve its catalytic activity and thermal stability, so as to help lay the foundation for its industrial application.

## 2. Results and Discussion

### 2.1. Screening of Modifiers

**Figure 1:**
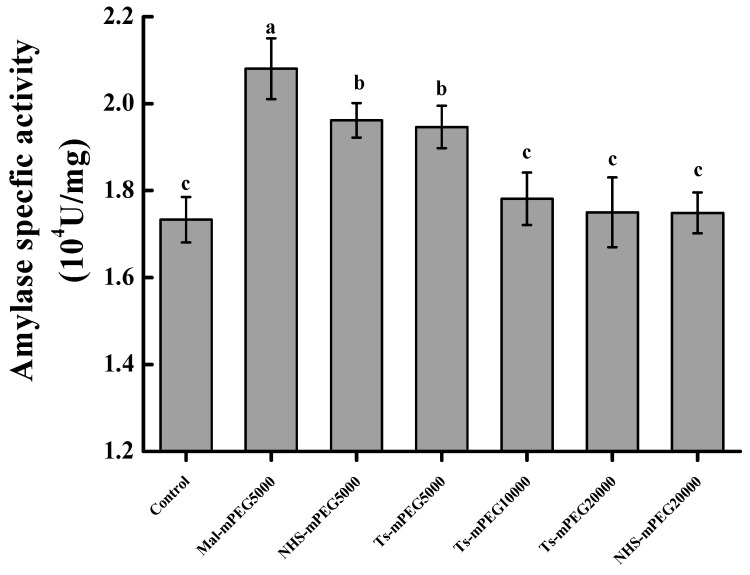
Effect of 6 types of modifiers on SPA activity. Significant difference in each column are expressed as different superscript letters (*p* < 0.05).

The change in SPA specific activity modified by 6 different modifiers was shown in Figure 1. Compared with the untreated SPA, Mal-mPEG5000, NHS-mPEG5000 and Ts-mPEG5000 could significantly increase SPA’s specific activity (*p* < 0.05) in the same molar ratio. The specific activity of Mal-mPEG5000-SPA reached the maximum value at (2.081 ± 0.050) × 10^4^ U/mg. There was a significant difference between Mal-mPEG5000 and other modifiers (*p* < 0.05). However, there was no significant difference between Ts-mPEG10000, Ts-mPEG20000 and NHS-mPEG20000 (*p* > 0.05). Therefore, Mal-mPEG5000 was selected as the optimal modifier for the next experiments.

### 2.2. Effect of the Molar Ratio on Modification

**Figure 2:**
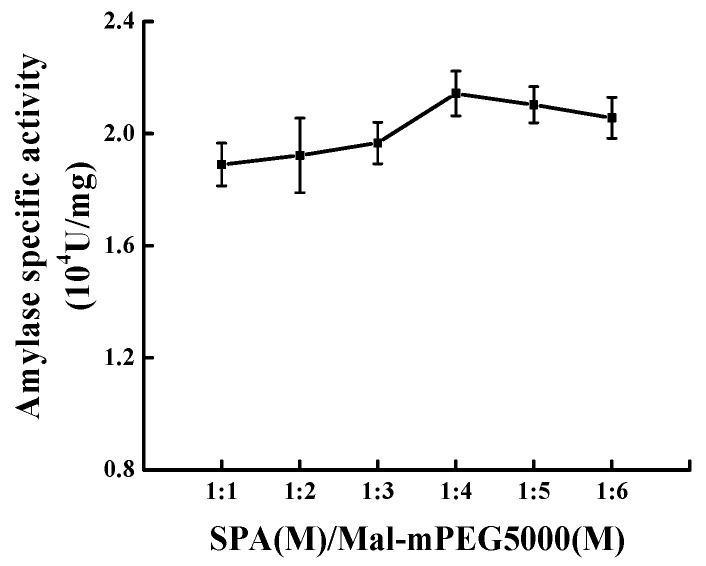
Effect of the molar ratio of SPA to Mal-mPEG5000 on SPA activity.

The results from Figure 2 indicated that as the molar ratio rose from 1:1 to 1:4, increasingly high SPA specific activity was found along with the increase of Mal-mPEG5000 concentration. The enzymatic specific activity reached the maximum (2.143 ± 0.050) × 10^4^ U/mg when the molar ratio was 1:4, an increase by 23.66% compared with that of the untreated SPA. As the concentration of Mal-mPEG5000 increased further, and the molar ratio increased to 1:6, the enzymatic specific activity decreased to (2.016 ± 0.051) × 10^4^ U/mg, and there was a significant difference between the molar ratio of 1:4 and 1:6 (*p* < 0.05). Therefore, the optimal response molar ratio of SPA to Mal-mPEG5000 was concluded to be 1:4.

### 2.3. Effect of Temperature on Modification

**Figure 3:**
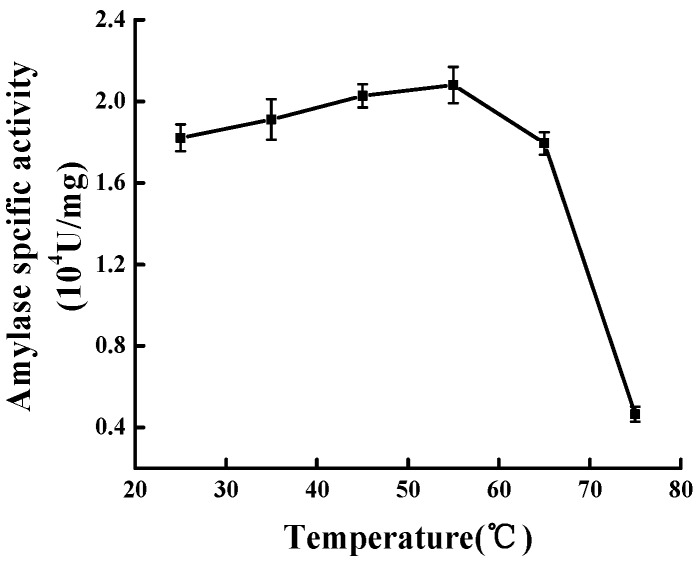
Effect of modification temperature on modification.

The results from Figure 3 indicated that the Mal-mPEG5000-SPA specific activity gradually increased as the temperature rose from 25 °C to 55 °C. The specific activity reached the maximum (2.131 ± 0.059) × 10^4^ U/mg at 55 °C. As the temperature increased to 65 °C, the specific activity dropped to (1.801 ± 0.055) × 10^4^ U/mg, and decreased by 15.49%, and there was a significant difference between 55 °C and 65 °C (*p* < 0.05). Therefore, the optimal modification temperature was concluded to be 55 °C.

### 2.4. Effect of pH on Modification

**Figure 4:**
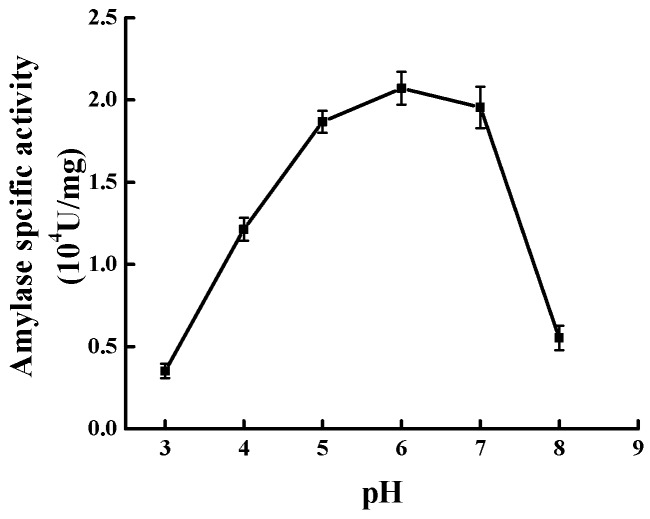
Effect of modification pH values on modification.

The results from Figure 4 indicated that the Mal-mPEG5000-SPA specific activity gradually increased as the pH moved from 3 to 6. The specific activity reached the maximum (2.155 ± 0.046) × 10^4^ U/mg at pH 6.0. As pH increased to 7.0, the specific activity was (1.925 ± 0.045) × 10^4^ U/mg and decreased by 10.69%, and there was a significant difference between pH 6.0 and 7.0 (*p* < 0.05). Therefore, the optimal modification pH was concluded to be 6.0.

### 2.5. Effect of Time on Modification

**Figure 5:**
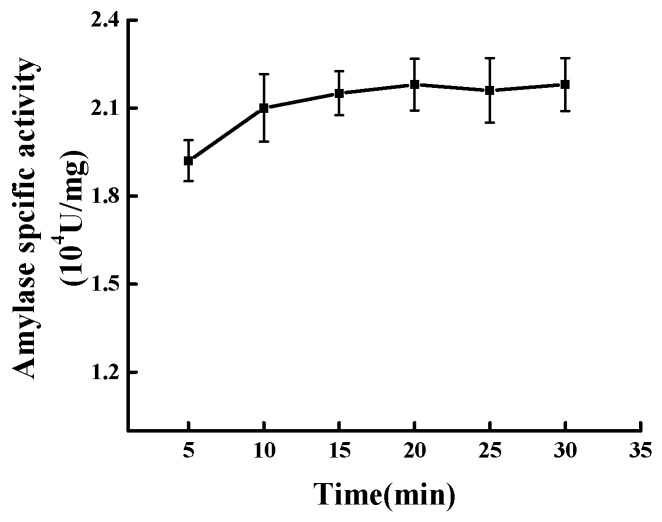
Effect of modification time on modification.

The results from Figure 5 indicated that the Mal-mPEG5000-SPA specific activity increased as the modification time rose from 5 min to 10 min. The specific activity reached the maximum (2.142 ± 0.059) × 10^4^ U/mg at 10 min. As the modification time further increased, no significant change was noted in the enzymatic specific activity (*p* = 0.05). The ANVOA results showed no significant difference of enzymatic specific activity between 10 min and 30 min. Therefore, taking into account of practical application, the optimal modification time was concluded to be 10 min.

### 2.6. Optimizing the Modification Procedure

According to the effect of the molar ratio, modification temperature, pH and time, the time has little effect on modification, and modification pH (A), the molar ratio (B) and modification temperature (C) were selected as three factors to optimize the modification procedure by central composite design (CCD). With modification time set at 10 min, and Mal-mPEG5000-SPA specific activity (Y) as the response value, Box-Behnken design principles were followed to conduct a test of three factors and three levels. Results were shown in Table 1.

Multiple regression analysis was adopted to the experimental data by using the software Design-Expert V8.0.6. The response, Y (Mal-mPEG5000-SPA specific activity), was selected as the test variables, and by the second order, a polynomial equation was developed (Equation (1)).

Y = 2.15 + 0.025A + 0.00528B + 0.016C − 0.033AB + 0.027AC + 0.0011BC − 0.17A^2^ − 0.13B^2^ − 0.13C^2^ (1)

The analysis of variance (ANOVA) for the response Y, the Mal-mPEG5000-SPA specific activity, was shown in Table 2. The regression model was highly significant (*p* < 0.01), while the lack of fit was not significant (*p* = 0.317 > 0.05) and the value of the determination coefficient (R^2^) was 0.996, which indicated the goodness of fit of the regression model [19]. Based on the analysis of experimental data in Table 1 and Table 2, the regression model demonstrated a high correlation, and can be used as theoretical prediction for the enzymatic specific activity of Mal-mPEG5000 modified SPA. The significance test on the regression model suggested that the effect on enzymatic specific activity was as follows: Modification pH > modification temperature > Molar ratio of SPA to Mal-mPEG5000.

**Figure 6:**
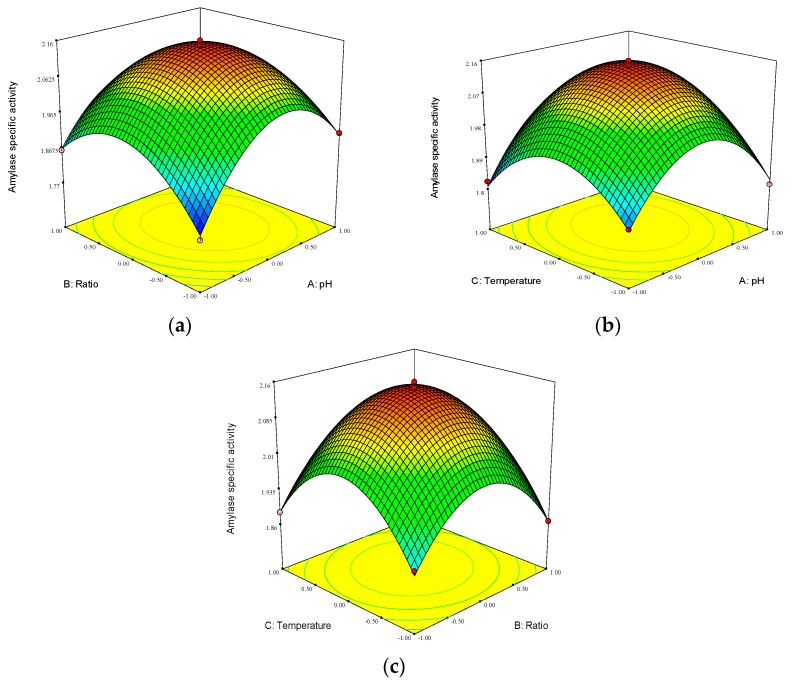
Response surface plot showing the effects of the variables on the activity of Mal-mPEG5000-SPA. The three independent variables set were modification pH (**a**), the molar ratio of SPA to Mal-mPEG5000 (**b**) and modification temperature (**c**).

In order to further comprehend the interaction between the parameters, the response surfaces were obtained using Equation (2), which were plotted between two independent variables and the other independent variable was set at the zero-coded level. The analysis on the response was shown in Figure 6a–c.

Figure 6a indicated that with the increase of the modification pH and the molar ratio, the Mal-mPEG5000-SPA specific activity first increased, and then declined, meanwhile, steep response surfaces and oval contour line were found, which indicated significant interaction of the pH value and the molar ratio.

Figure 6b suggested that with the increase of modification pH and temperature, the Mal-mPEG5000-SPA specific activity firstly increased, and then decreased, and the oval contour line was noticed, which demonstrated a significant interaction of modification pH value temperature.

Figure 6c revealed that with the increase of the molar ratio and modification temperature, the Mal-mPEG5000-SPA specific activity increased firstly before it declined, and the occurrence of circular contour line showed no significant interaction of the molar ratio and modification temperature.

Through the analysis by the software Design-Expert, the optimized combination for maximum Mal-mPEG5000-SPA specific activity was determined through canonical analysis of the response surfaces and contour plots as A = 6.08, B = 4.04 and C = 58.85 °C, i.e., the predicted value of Mal-mPEG5000-SPA specific activity of 2.247 × 10^4^ U/mg. under modification pH 6.08, the molar ratio 1:4.04, and modification temperature 58.85 °C. Considering the practicality of the validation test, the optimum parameters were corrected into modification pH 6.0, the molar ratio 1:4, and modification temperature 58 °C. The specific activity of Mal-mPEG5000-SPA was determined under the corrected parameters to be (2.150 ± 0.055) × 10^4^ U/mg, an increase by 24.06% than that of the untreated one. The validation test showed that the optimization results from the response surface method were reliable, and adopting response surface method to optimize the modification process of Mal-mPEG5000 on SPA was feasible.

### 2.7. Separation and Purification of Mal-mPEG5000-SPA

**Figure 7:**
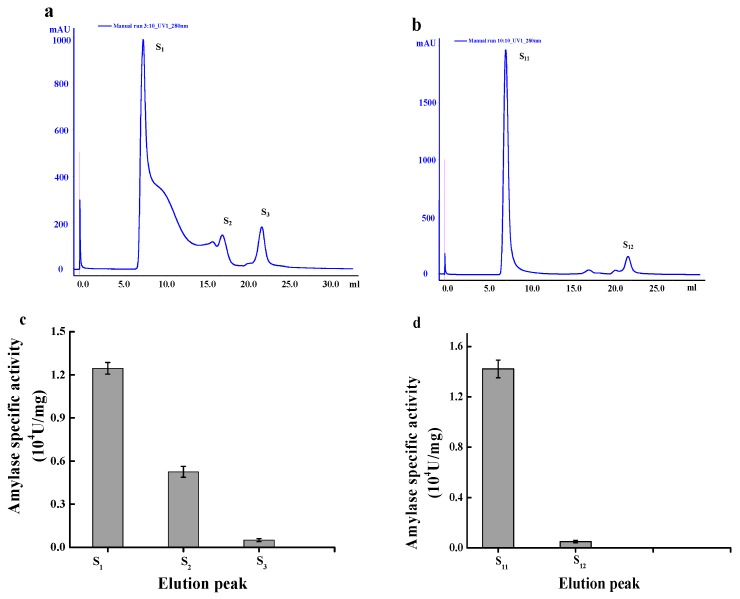
Separation curve of Mal-mPEG5000-SPA by gel column Superdex^TM^75 (**a**)Elution profile of Mal-mPEG5000-SPA by Superdex^TM^75 for the first time; (**b**) Elution profile of Mal-mPEG5000-SPA by Superdex^TM^75 for the second time; (**c**) The specific activity of the elution fractions from Superdex^TM^75 for the first time; (**d**) The specific activity of the elution fractions from Superdex^TM^75 for the second time.

Mal-mPEG5000-SPA was separated and purified by AKTA purifier^TM^10 with Superdex^TM^75 gel column (Figure 7a–c). Separated by Superdex^TM^75 gel column, there were three protein peaks: S_1_, S_2_ and S_3_ (Figure 7a), and the eluents in the collection tubes corresponding to the peak position were collected and determined for enzymatic specific activity. The specific activity of S_1_ and S_2_ was (1.245 ± 0.047) × 10^4^ U/mg and (0.533 ± 0.036) × 10^4^ U/mg respectively as shown in Figure 7c. As the peak of SPA elution was noted around 17 min according to the result of the pre-test, the elution peak S_1_ was collected, centrifuged and concentrated for further purification.

Mal-mPEG5000-SPA was separated for the second time using Superdex^TM^75 gel column, and there were elution peaks S_11_ and S_12_ (Figure 7b). The specific activity of S_11_ and S_12_ was determined to be (1.422 ± 0.057) × 10^4^ U/mg and (0.061 ± 0.009) × 10^4^ U/mg respectively (Figure 7d). According to the separation principles of gel column chromatography, different positions of the peaks suggest different molecular weight of the protein under each peak. The position of SPA peak was around 17 min, while the position of Mal-mPEG5000-SPA was about 7 min, which indicated change in molecular weight as SPA was modified by Mal-mPEG5000.

**Figure 8:**
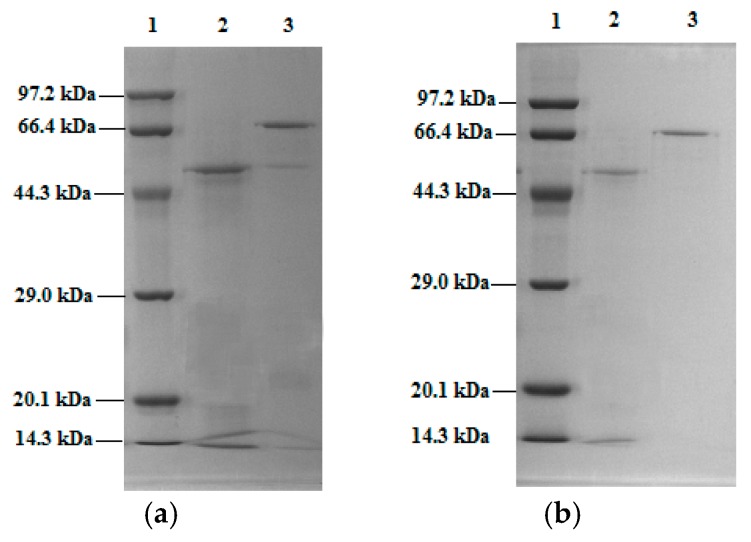
SDS-PAGE electrophoresis spectra of Mal-mPEG5000-β-SPA (**a**) separation by Superdex^TM^75 for the first time; Lane 1: marker proteins; Lane 2: SPA; Lane 3: Mal-mPEG5000-SPA by Superdex^TM^75. (**b**) separation by Superdex^TM^75 for the second time; Lane 1: marker proteins; Lane 2: SPA; Lane 3: Mal-mPEG5000-SPA by Superdex^TM^75.

SDS-PAGE gel electrophoresis was carried out on the collected elution peaks S_1_ and S_11_. The results were shown in Figure 8a,b. An obvious band of S_1_ was found above the SPA band as indicated in Figure 8a. The band was of Mal-mPEG5000-SPA, whose molecular weight was about 67 kDa compared with the standard marker. Figure 8b demonstrated the electrophoretogram of elution peak S_11_. A single band was noted after separation twice by Superdex^TM^75, and Mal-mPEG5000-SPA was obtained through purification.

### 2.8. Optimum Temperature and Thermal Stability of Mal-mPEG5000-SPA and SPA

**Figure 9:**
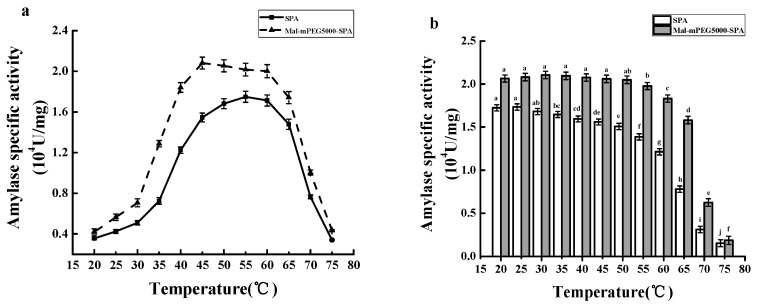
Temperature profiles of Mal-mPEG5000- SPA and SPA. (**a**) Optimum enzymolysis temperature; (**b**) Thermal stability. Significant difference in each column are expressed as different superscript letters (*p* < 0.05).

According to Figure 9a, the maximum specific activity of SPA was found to be (1.733 ± 0.050) × 10^4^ U/mg at 55 °C, while the maximum of Mal-mPEG5000-SPA was (2.121 ± 0.058) × 10^4^ U/mg at 45 °C, an increase by 22.38% compared to the untreated one. Moreover, the activities of Mal-mPEG5000-SPA at 50 °C, 55 °C and 60 °C were determined as (2.074 ± 0.060) × 10^4^ U/mg, (2.057 ± 0.062) × 10^4^ U/mg, and (2.031 ± 0.064) × 10^4^ U/mg respectively, all significantly higher than that of untreated SPA (*p* < 0.05). These results indicated that the enzymatic specific activity of modification enzyme was significantly improved after Mal-mPEG5000 modification.

Figure 9b showed the thermal stability of Mal-mPEG5000-SPA and SPA. No significant difference in SPA specific activity at 20 °C and 25 °C for 1 h was noted. However, when the temperature rose to 30 °C, the specific activity of SPA was significantly reduced (*p* < 0.05), which indicated high sensitiveness of SPA to temperature. No significant difference in the specific activity of Mal-mPEG5000-SPA from 20 °C to 45 °C for 1 h was found. But when the temperature rose to 50 °C, the specific activity of Mal-mPEG5000-SPA started to decline (*p* < 0.05), which suggested significant improvement of the thermal resistance of Mal-mPEG5000-SPA. These results showed that hydrolyzed starch by Mal-mPEG5000-SPA has a wider range of application in the food industry, which can significantly enhance enzymatic efficiency to reduce production costs.

### 2.9. Optimum pH and pH Stability of Mal-mPEG5000-SPA and SPA

**Figure 10:**
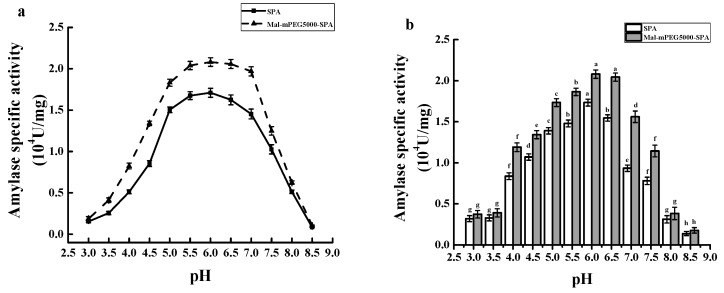
pH profiles of Mal-mPEG5000- SPA and SPA. (**a**) Optimum enzymolysis pH; (**b**) pH stability. Significant difference in each column are expressed as different superscript letters (*p* < 0.05).

Figure 10a showed that Mal-mPEG5000-SPA and SPA both showed relatively high specific activity from pH 5.0 to 7.0. At pH 6.0, the specific activity of Mal-mPEG5000-SPA and SPA reached respective maximum values at (2.061 ± 0.051) × 10^4^ U/mg and (1.733 ± 0.050) × 10^4^ U/mg, and the specific activity was improved by 18.92% after modification.

Figure 10b showed the pH stability of Mal-mPEG5000-SPA and SPA. A significant difference in SPA’s specific activity at pH 6.0 and 6.5 was found (*p* < 0.05), while the difference for Mal-mPEG5000-SPA was not significant (*p* = 0.05), which indicated higher adaptability of Mal-mPEG5000-SPA to a pH environment. In addition, the specific activity of Mal-mPEG5000-SPA was constantly higher than that of SPA under pH value from 4.0 to 7.5, which indicated lower susceptibility of Mal-mPEG5000-SPA to pH environment. This means Mal-mPEG5000-SPA could applied more broadly and is more suitable for industrial application.

### 2.10. Kinetic Parameters of Mal-mPEG5000-SPA

The results of kinetic parameters of Mal-mPEG5000-SPA were shown in Table 3, the K_m_ value for sweet potato starch, potato starch, corn starch, soluble starch, amylase and amylopectin by Mal-mPEG5000-SPA hydrolysis was respectively (1.63 ± 0.033) mg/mL, (2.06 ± 0.028) mg/mL, (2.36 ± 0.063) mg/mL, (1.84 ± 0.025) mg/mL, (2.18 ± 0.029) mg/mL and (2.11 ± 0.052) mg/mL. The V_max_ value was respectively (32.06 ± 0.61) mmol/min/mL, (16.23 ± 0.32) mmol/min/mL, (10.66 ± 0.37) mmol/min/mL, (20.88 ± 0.78) mmol/min/mL, (13.35 ± 0.38) mmol/min/mL, (13.89 ± 0.41) mmol/min/mL. The *E_a_* value was respectively (11.07 ± 0.43) kJ/mol, (18.24 ± 1.12) kJ/mol, (26.52 ± 1.21) kJ/mol, (14.71 ± 1.15) kJ/mol, (21.16 ± 1.12) kJ/mol and (21.66 ± 0.8) kJ/mol. These results indicated that Mal-mPEG5000-SPA had the lowest Michaelis constant for sweet potato starch. Therefore, Mal-mPEG5000-SPA was determined to have the strongest binding affinity for sweet potato starch. Next only to sweet potato starch, soluble starch was followed by potato starch, amylase and amylopectin. Compared with the SPA kinetic parameters reported in earlier studies [20], for sweet potato starch hydrolyzed by Mal-mPEG5000-SPA, K_m_ declined by 12.95%, V_max_ increased by 26.87%, and *Ea* dropped by 12.63%, which suggested Mal-mPEG5000-SPA showed stronger affinity for sweet potato starch than SPA.

### 2.11. Effect of Metal Ions on the Activity of Mal-mPEG5000-SPA

As shown in Table 4, the relative specific activity after addition of Mn^2+^, K^+^, Zn^2+^ and Ca^2+^ was respectively (143.48 ± 6.25)%, (115.49 ± 4.51)%, (111.28 ± 4.82)% and (102.88 ± 4.32)%, which demonstrated that Mn^2+^, K^+^, Zn^2+^ and Ca^2+^ had good activation effect. Mg^2+^ showed a slight inhibitive effect on Mal-mPEG5000-SPA, with relative specific activity standing at (95.25 ± 4.14)%. However, Cu^2+^, NH_4_^+^, Fe^3+^, Al^3+^, Ba^2+^ and EDTA demonstrated a relatively strong inhibitory effect on Mal-mPEG5000-SPA, with relative specific activity reduced to 55–80%. Meanwhile, under the effect of Cd^2+^, Hg^+^ and Ag^+^, the relative specific activity were all below 30%, among which Hg^+^ and Ag^+^ indicated the strongest inhibitory effect on Mal-mPEG5000-SPA. Compared with the effect of metal ions on SPA reported in earlier studies [21], the effect of metal ions on Mal-mPEG5000-SPA and on SPA showed no significant difference (*p* = 0.05).

## 3. Materials and Methods

### 3.1. Materials

Sweet potato, molar weight 56.043 kDa, harvested in Xinxiang City, Henan Province, China, its cultivar was Shangshu 19. NHS-mPEG5000, NHS-mPEG20000, Ts-mPEG5000, Ts-mPEG10000, Ts-mPEG20000 and Mal-mPEG5000 were from Nanocs (New York, NY, USA; purity ≥ 95%). All other reagents were of analytical reagent grade and used without further purification.

### 3.2. Separation and Purification of SPA

Using the methods described by Liang et al. [21] with minor modification. SPA was separated and purified with the following steps. Fresh sweet potatoes were washed and sliced into chips. 100 g sweet potatoes were weighed out, and 200 mL distilled water was added for pulverization in a pulverizer (SQ2002, Shanghai Shuaijia Electronic Technology Company, China) for 1 min. The sample was filtered through 40 mesh sieve, and the resulting filtrate was placed in a refrigerated centrifuge at 4 °C for centrifugation at 4000 rpm for 15 min. The supernatant was obtained, and ammonium sulfate was added to the supernatant to 70% saturation. The resulting solution was stored in a refrigerator at 4 °C for 4 h. The centrifuge parameters were set at 4 °C and 8000 rpm for refrigerated centrifugation for 15 min. The resulting precipitate was collected, and dissolved in a bufferA. Ammonium sulfate was desalinated by using 1 kDa ultrafiltration membrane for 4 h. Protein purifier (AKTA purifier^TM^10, General Electric Company, Boston, Massachusetts, MA, USA) was used to purify the enzyme, and Mono Q anion exchange chromatographic column and Superdex^TM^75 gel column were adopted for separation and purification with detection wavelength set at 280 nm. The buffers for purification were as follows. Buffer A: 20 mM pH 5.8 disodium-hydrogen phosphate-citric acid, Buffer B: 1 mol/L NaCl. The buffers were filtered through a 0.22 μm membrane, ultrasound treated for 20 min, and stored in a refrigerator at 4 °C. Buffer A was used to equilibrate the chromatographic column. The sample was injected after equilibration of buffer to the baseline. The column was washed with 5 column volumes of buffer A, and then eluted with a gradient from 0–100% Buffer B at the flow rate of 1.0 mL/min, and maximum back-pressure of 4 MPa for 30 min. A collector was used to obtain 1 mL fractions. After the elution was complete, the enzymes in the collection tube corresponding to the peak position were collected, frozen and dried in a vacuum freeze dryer (Thermo Savan, Thermo Electron Co., Waltham, MA, USA) and stored at 4 °C for later usage. The separation and purification of mPEG5000-β-SPA: 70% ammonium sulfate was added into the reaction liquid to precipitate. The rest steps were the same as those of the β-amylase separation and purification.

The molecular mass and the purity of the enzyme was conducted by SDS-PAGE according to the Laemmli [22] method, and 15% (*w*/*v*) polyacrylamide gel was adopted.

### 3.3. Protein Content and Enzyme Assay

Protein content was measured as described by Lowry et al. [23], and bovine serum albumin was used as the standard. Enzyme assay was determined by the dinitrosalicylic acid (DNS) method according to Sagu et al. [24] with minor modification. The enzyme assay was performed at 40 °C, pH 5.8, and 1 mg maltose released per hour from 1.1% soluble starch was defined as a unit of enzyme specific activity. In this paper, the enzyme activity was indicated by specific enzyme activity, expressed as U/mg.

### 3.4. Screening of Modifiers

The molar ratio of SPA to modifiers was determined at 1:4. SPA and 6 different types of modifiers (NHS-mPEG5000, NHS-mPEG20000, Ts-mPEG5000, Ts-mPEG10000, Ts-mPEG20000 and Mal-mPEG5000) were placed in the buffer (disodium-hydrogen phosphate-citric acid acid, pH 6.0), respectively, and then kept in a water-bath thermostatic metal oscillator at 55 °C for 10 min. The reaction mixture was obtained for dialysis. After the dialysis was complete, the enzyme specific activity was measured to obtain the optimal modifier.

### 3.5. Selection of Relevant Variables and Experimental Design

SPA was modified by Mal-mPEG5000 at the molar ratio of SPA to Mal-mPEG5000 from 1:1 to 1:6, for modification temperature from 25 °C to 75 °C and for modification pH from 3.0 to 8.0. The enzyme specific activity of modified enzyme (expressed as Mal-mPEG5000- SPA) was used as the index for evaluating the effects of the different modification parameters to be optimized via response surface methodology (RSM). A central composite design (CCD) were used with three variables and three levels for optimizing the modification conditions. These three factors were modification pH (A), the molar ratio of SPA to Mal-mPEG5000 (B) and modification temperature (C) (Table 5). A second-order polynomial equation (Equation (2)) for the variables was developed:

Y = α_0_ + α_1_A + α_2_B + α_3_C + α_11_A^2^ + α_22_B^2^ + α_33_C^2^ + α_12_AB + α_13_AC + α_23_BC (2)

Y is the predicted response, α_0_ is the intercept; α_1_, α_2_, α_3_, linear coefficients; α_11_, α_22_, α_33_, squared coefficients; and α_12_, α_13_, α_23_, interaction coefficients. Analysis of variance was used to evaluate the model’s adequacy and to determine the regression coefficients and their statistical significance. The response surface contour plots showed how the independent variables interacted and how those interactions influenced the overall response [20].

### 3.6. Enzyme Characterization

Influence of temperature on Mal-mPEG5000-SPA and SPA: The optimum temperature of Mal-mPEG5000-SPA was investigated at pH 5.8 over a temperature range of 20–75 °C. SPA and Mal-mPEG5000-SPA was incubated in 20 mM disodium-hydrogen phosphate-citric acid buffer (pH 5.8) during 60 min, respectively. The thermostability of the enzyme residual specific activity were determined under different temperature conditions (20–75 °C).

Influence of pH on Mal-mPEG5000-SPA and SPA: The optimum pH of Mal-mPEG5000-SPA was investigated at 50 °C over a pH range of 3.0–8.5. Mal-mPEG5000-SPA was preincubated for 60 min under 50 °C, the residual specific activity was determined at a pH range of 3.0–8.5 to evaluate the pH stability.

### 3.7. Effect of Metal Ions on Mal-mPEG5000-SPA and SPA

Incubation of the enzyme was carried out by 10 mM of metal ions salts (chlorides including Ca^2+^, Mg^2+^, Cu^2+^, Zn^2+^, Mn^2+^, K^+^, NH^4+^, Hg^+^, Al^3+^, Fe^3+^, Ba^2+^ and Cd^+^), AgNO_3_ and EDTA at 40 °C for 30 min, and the residual activities were determined respectively. An enzyme that did not contain metal ions was chosen as control (100%).

### 3.8. Kinetic Constant

The Michaelis constant (K_m_) and the maximum velocity (V_max_) of Mal-mPEG5000-SPA and SPA was defined by Lineweaver-Burk plot. Different concentrations of substrates (1–20 mg/mL) in 20 mM disodium-hydrogen phosphate-citric acid buffer pH 5.8 were used and specific activity was assessed by the DNS method. The values of K_m_ and V_max_ were calculated respectively based on the double reciprocal plot [25].

### 3.9. Activation Energy (Ea)

*E_a_* of the enzyme was measured by Arrhenius equation (lnk_cat_ = lnk_0_ − *Ea*/RT), and the temperature was set from 25 °C to 65 °C. Representing 1/T in K on the axis of x and natural log of specific activity on the axis of y, Arrhenius plot was generated. *E_a_* was defined based on the value of the slope.

### 3.10. Statistical Analysis

All experiments were carried out in triplicate. SPSS 22.0 statistical software (SPSS Inc., Chicago, IL, USA) was used for the analysis of variance, and the significance test (*p* < 0.05) was performed by Duncan new complex method. All analyses were done in triplicate, and the statistical significance was determined using the mean values ± standard deviation.

## 4. Conclusions

In this study, we selected Mal-mPEG5000 as the best modifier from six modifiers and optimized the modification parameters by the response surface method. We used column chromatography to isolate and purify Mal-mPEG5000-SPA from the reaction solution between SPA and Mal-mPEG5000. Thereafter, the enzymatic properties were determined and compared in the presence and absence of Mal-mPEG5000. For the results, under the optimal conditions (the molar ratio of Mal-mPEG5000 to SPA, 1:4, modification temperature, 58 °C, pH 6.0), the specific activity of Mal-mPEG5000-SPA was (2.150 ± 0.055) × 10^4^ U/mg, an increase by 24.06% than that of the unmodified SPA. Mal-mPEG5000-SPA was separated and purified, and a single band was noticed and its molecular weight was about 67 kDa. The enzyme properties of Mal-mPEG5000-SPA were significantly improved as the optimum temperature declined from 55 °C to 45 °C, the thermal stability and pH stability were obviously enhanced; the K_m_ value of sweet potato starch by Mal-mPEG5000-SPA declined by 12.95%, while V_max_ increased by 26.87%, and Ea dropped by 12.63%, which showed that Mal-mPEG5000-SPA had greater affinity for sweet potato starch and higher the speed of hydrolysis than SPA; as for Mal-mPEG5000-SPA, Mn^2+^, K^+^, Zn^2+^ and Ca^2+^ demonstrated activation effect; Mg^2+^ showed a slight inhibitory effect; Cu^2+^, NH_4_^+^, Fe^3+^, Al^3+^, Ba^2+^, EDTA, Hg^+^ and Ag^+^ had a relatively strong inhibitory effect; there was no significant difference of metal ions’ effect on Mal-mPEG5000-SPA and SPA. It is concluded that Mal-mPEG5000-SPA will have a wider range of application in beer processing, maltose production and other food industries. Therefore, the studies on SPA application in food industries are theoretically and practically significant.

## Figures and Tables

**Table 1:** Experimental design and results for the activity of Mal-mPEG5000-SPA.

Runs		Coded Levels		Response Value
	pH	Molar Ratio	Temperature (°C)	(×10^4^ U/mg)
1	−1	−1	0	1.7763
2	0	−1	−1	1.8858
3	0	0	0	2.1592
4	0	0	0	2.1481
5	1	0	−1	1.8142
6	0	1	−1	1.8667
7	0	−1	1	1.8758
8	1	−1	0	1.9097
9	0	0	0	2.1323
10	−1	1	0	1.8620
11	−1	0	1	1.8226
12	0	0	0	2.1597
13	0	0	0	2.1326
14	0	1	1	1.9097
15	1	0	1	1.9097
16	1	1	0	1.8619
17	−1	0	−1	1.8362

**Table 2:** Analysis of variance (ANOVA) for the experimental results.

Source	Sum of Squares	df	Mean Square	F-Value	*p*-Value	Significance
Model	0.3	9	0.034	145.34	<0.0001	**
A	4.91 × 10^−3^	1	4.91 × 10^−3^	21.05	0.0025	**
B	2.23 × 10^−^^4^	1	2.23 × 10^−^^4^	0.96	0.3606	
C	1.95 × 10^−3^	1	1.95 × 10^−3^	8.36	0.0233	*
AB	4.48 × 10^−3^	1	4.48 × 10^−3^	19.23	0.0032	**
AC	2.98 × 10^−3^	1	2.98 × 10^−3^	12.76	0.0091	**
BC	4.62 × 10^−^^4^	1	4.62 × 10^−^^4^	1.98	0.2022	
A2	0.12	1	0.12	507.79	<0.0001	**
B2	0.067	1	0.067	288.2	<0.0001	**
C2	0.074	1	0.074	319.52	<0.0001	**
Residual	1.63 × 10^−3^	7	2.33 × 10^−^^4^			
Lack of fit	8.97 × 10^−^^4^	3	2.99 × 10^−^^4^	1.63	0.317	
Pure error	7.35 × 10^−^^4^	4	1.84 × 10^−^^4^			
Cor total	0.31	16				
R2	0.996					

* indicate significant (*p* < 0.05); ** indicate highly significant (*p* < 0.01).

**Table 3:** Kinetic and activation energy parameters of Mal-mPEG5000-SPA.

Substrates	K_m_ (mg/mL)	V_max_ (mmol/min/mL)	*E_a_* (kJ/mol)
Sweet potato starch	1.87 ± 0.032 ^a^	19.32 ± 0.65 ^e^	12.67 ± 0.73 ^a^
Potato starch	2.16 ± 0.031 ^c^	14.62 ± 0.35 ^c^	19.84 ± 1.03 ^c^
Corn starch	2.56 ± 0.053 ^e^	9.36 ± 0.27 ^a^	28.23 ± 1.37 ^e^
Soluble starch	1.96 ± 0.029 ^b^	16.75 ± 0.68 ^d^	16.61 ± 0.99 ^b^
Amylase	2.32 ± 0.037 ^d^	12.85 ± 0.42 ^b^	22.26 ± 1.09 ^d^
Amylopectin	2.37 ± 0.045 ^d^	12.53 ± 0.41 ^b^	22.54 ± 0.98 ^d^

Significant difference in each column are expressed as different superscript letters (*p* < 0.05).

**Table 4:** Effect of metal ions on the activity of Mal-mPEG5000-SPA.

Metal Ions	Relative Activity (%)
Control	100
Ca^2+^	102.88 ± 4.32 ^c^
Mg^2+^	95.25 ± 4.14 ^d^
Cu^2+^	60.95 ± 3.61 ^f^
Zn^2+^	111.28 ± 4.82 ^b^
Mn^2+^	143.48 ± 6.25 ^a^
K^+^	115.49 ± 4.51 ^b^
NH_4_^+^	78.89 ± 4.05 ^e^
Hg^+^	5.21 ± 0.63 ^i^
Ag^+^	3.28 ± 0.12 ^i^
Al^3+^	45.81 ± 3.52 ^g^
Fe^3+^	80.85 ± 4.62 ^e^
Ba^2+^	59.68 ± 3.92 ^f^
Cd^2+^	22.85 ± 2.06 ^h^
EDTA	55.84 ± 3.27 ^f^

Significant difference in each column are expressed as different superscript letters (*p* < 0.05).

**Table 5:** Independent variables and levels for central composite design (CCD).

Factors	Levels		
	−1	0	1
Modification pH	5.0	6.0	7.0
Molar ratio of SPA to Mal-mPEG5000	1:3	1:4	1:5
Temperature/°C	45	55	65
